# Undergraduate Interprofessional Education: Protocol for a Mixed Methods Study

**DOI:** 10.2196/74394

**Published:** 2026-04-08

**Authors:** Hannah Snead, Dhwaniben Hada, Lori Ann Eldridge

**Affiliations:** 1 East Carolina University, 3204 Carol G. Belk Building, Greenville, NC, 27858, United States, 1 252-328-2986

**Keywords:** interprofessional education, undergraduate, health care, self-efficacy, protocol

## Abstract

**Background:**

Interprofessional education (IPE) is essential for fostering collaboration and communication among health care professionals to improve patient outcomes. Early IPE training for prehealth undergraduate students has been shown to enhance teamwork, dismantle stereotypes, and build self-efficacy in health care settings. Self-efficacy is the belief that one can achieve their goals and succeed at tasks. These outcomes contribute to creating a cohesive and equitable health care environment focused on patient-centered care. Despite evidence supporting the benefits of IPE training, there is a lack of consistent implementation at the undergraduate level across institutions.

**Objective:**

Programs that have adopted IPE have demonstrated improved student preparedness, better interprofessional collaboration, and enhanced patient care outcomes. Our study addresses this gap in undergraduate IPE among institutions by examining the impact of a structured IPE training program, Pathway to Health Professions, designed for prehealth East Carolina University undergraduate students who are members of Buff in Scrubs, an East Carolina University organization.

**Methods:**

Our study uses a mixed methods approach, including retrospective surveys and open-ended questions, to assess changes in participants’ attitudes, skills, and competencies before and after training. Data will be collected from January 2025 to April 2025.

**Results:**

We hypothesize that early and structured exposure to IPE training will significantly enhance prehealth undergraduate students’ confidence, understanding of interprofessional roles, and ability to collaborate effectively. By focusing on self-efficacy–based skills, we hope to equip prehealth students with the confidence to apply to their desired health professions program.

**Conclusions:**

This paper outlines the protocol for Pathway to Health Professions, a prehealth preparation IPE program. Future testing of the use of this program is outlined. We hypothesize that the results from the future testing of this program will show an improvement in undergraduate prehealth students’ self-efficacy related to graduate school readiness.

## Introduction

### Background

Health care is a diverse field that encompasses many occupations collaborating to provide patient care. It is important for future health care workers to be aware and informed of how to interact with one another to have a productive health care team. Our program, Pathway to Health Professions, is based on current research regarding the best interprofessional education (IPE) content and practices. This IPE program is intended to equip prehealth students with different skills to help prepare them for their future careers. Some of these skills include but are not limited to teamwork, leadership, collaboration between health care disciplines, networking, and decision-making. This program hopes to inspire prehealth undergraduates to invest in their future health care occupations and learn about IPE within the health care field. IPE involves developing and structuring programs to educate and facilitate collaboration across various health care professions [1]. It focuses on collaboration and communication between professionals for better-working teams [2]. Multiple studies have demonstrated that the early implementation of IPE training for undergraduate students can have a positive impact on teamwork, collaboration, and confidence [3].

Studies have found that introducing IPE before starting graduate training programs enhances the understanding of various health professions [4]. IPE training helps dismantle stereotypes that many prehealth students and professionals hold about health care teams [5,6]. Eliminating these stereotypes and receiving information on the role of each profession creates a more cohesive health care environment for all health care professionals to work together [7]. Early focus on teamwork and collaboration can create more responsive students who can better adapt to group environments, which affects patient care and outcomes [8,9]. IPE training can focus on caring for vulnerable populations and ensure equitable care [10]. Not only is equitable care an outcome of IPE training, but it has also been shown that the use of IPE training creates a more comprehensive team that further promotes better health outcomes and shorter hospital stays for patients [11]. Furthermore, it has been shown that IPE training offers students a safe environment in which to practice caring for patients. This overall improves predicted patient outcomes and encourages confidence in students [12]. Major universities and medical programs have recognized the gaps present and the outcomes of IPE training and have begun implementing IPE training into their programs to bridge these gaps and prevent lapses in care [13]. Subsequently, IPE training has been successfully implemented in specific medical programs, yielding positive outcomes [14]. Those programs that have implemented IPE training have observed better graduate outcomes in students who completed it, as well as an overall increase in positive health care environments [15]. However, despite the success that several medical programs have experienced, many others have not incorporated IPE training [16].

Studies suggest that implementing IPE training sessions at the undergraduate level can address the improvement in teamwork, mutual respect, and patient-centered care approaches required by upper-level programs [17]. This has been supported by multiple studies that show the outcomes of implementing IPE programs [18]. In one study, multiple prehealth students focused on different professions were brought together. They participated in a short IPE training in which collaboration and education were a focus. After the study, students felt more competent and confident in their leadership skills and felt that they could better understand their interprofessional coworkers [3]. Another cross-sectional study of students who completed surveys while in their undergraduate and postgraduate programs found that identity and teamwork were affected by involvement in IPE events [15]. These short IPE studies show the need for longer programs with multiple sessions focusing on early intervention for prehealth undergraduate students. Furthermore, the most effective IPE training is multidisciplinary and uses various teaching methods [3]. With the increased interest in the continuation and implementation in universities and higher education programs, IPE training programs with the mission to educate prehealth students will become more common in the future [19].

### IPE Training

IPE refers to the education and overall design of programs that focus on the education and facilitation of learning among different health care professions. This is especially important in the health care field, where people from different occupations collaborate to treat patients. Given the variations among patients, it is best for the health care team to operate smoothly for fast and reliable care [2].

A meta-study by Spaulding et al [1] examined 19 articles to determine the benefits of exposing health care students to other health care fields. Attitudes toward other disciplines and the value placed on a team-based approach were found to improve statistically in 17 of the 19 studies. Due to the importance of IPE in the health care field, many universities now require IPE-specific health accreditation standards for disciplines such as dentistry, medicine, nursing, occupational therapy, pharmacy, physical therapy, physician assistant studies, and public health [20]. Spaulding et al [1] identified 7 studies that demonstrated statistically significant improvements in collaborative behavior through IPE. Another study examined whether the introduction of IPE influenced students’ perceptions of certain occupations. The study found that students who participated in IPE activities exhibited significant changes in behavior and attitudes toward other occupations [21]. While exposure to collaborative environments in health care education fosters improved interdisciplinary attitudes and behaviors, another critical factor influencing student success is self-efficacy.

Significant research has been dedicated to improving undergraduate students’ confidence, which has been linked to an overall enhancement in their professional experiences. A study specifically focused on preparing medical students for the health field found that having a strong professional identity and effective collaboration skills was important for future success [15]. Another study examined the relationship between health care and IPE centered on participant impact [4]. During students’ clinical rotations, they received IPE training and were later surveyed about their experiences. The findings revealed that introducing IPE before students started their programs enhanced their understanding of various health professions [4]. This literature indicates that self-efficacy and IPE may enhance one another and offer better support to prehealth students.

### Self-Efficacy: Comparison With Prior Work

Prior interventions to prepare prehealth students or early-stage trainees often focus on a single component, such as mentoring, shadowing, standardized patient encounters, or graduate program application workshops, and are typically delivered as stand-alone workshops or brief series. In contrast, the Pathway to Health Professions program integrates these elements into a coherent, longitudinal, IPE-focused program (Multimedia Appendix 1).

Session 1 (“Mentor Match Up”) extends existing mentoring models by pairing undergraduates with graduate-level health profession students, providing near-peer guidance that may be particularly salient for first-generation students and underrepresented students, such as those from rural communities; women; Black, Latino, and Native American students; and students with a low socioeconomic status. Session 2 (“Rounds Revealed”) adapts clinical reflection and ward-round models to an undergraduate context to demystify clinical environments and normalize early professional socialization. Session 3 (“Medical Mock Trials”) builds on simulation and standardized patient approaches to foster communication skills, role clarity, and critical thinking in interprofessional teams. Session 4 (“Equity in Action”) explicitly centers health equity and social determinants of health, which are often underemphasized in prehealth curricula despite their relevance to graduate competencies. Sessions 5 and 6 (“Pathway Prep” and “Health Roles Unveiled”) respond to literature documenting confusion and anxiety about competitive graduate program application processes and limited understanding of diverse health careers by offering structured, concrete guidance on applications, interviews, and interprofessional roles.

By linking these evidence-informed components into a sequenced curriculum, this program seeks to address multiple domains of self-efficacy simultaneously, academic and professional preparedness, networking, communication, equity-focused practice, and role clarity rather than targeting any single skill in isolation.

### Study Purpose and Objectives

An IPE-based training program called Pathway to Health Professions was developed to further support prehealth undergraduate students and prepare them for higher-level graduate programs. The Pathway to Health Professions program (n=6 sessions) will be tested to determine whether it was effective in improving self-efficacy regarding graduate school for undergraduate students in prehealth majors. We hypothesize that prehealth students who engage in all 6 Pathway to Health Professions IPE sessions will have improved self-efficacy regarding graduate training programs. This program differs from those in previous studies in that it spans a longer period and has multiple sessions addressing various aspects of health care IPE.

## Methods

### Study Design: Theory

**Figure 1.**
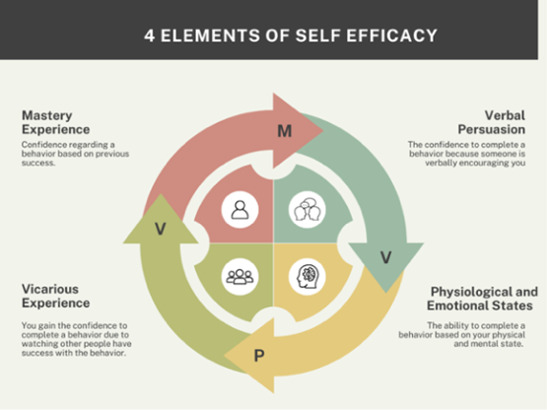
The 4 elements of self-efficacy.

In 1984, Bandura [22] introduced the theory of self-efficacy, which described the relationship between confidence and motivation. It refers to the fact that people are more likely to complete a task or move forward with a goal if they believe they have the capability to do so. Self-efficacy theory has 4 main components: experience, vicarious experience, social persuasion, and physiological feedback [22] (Figure 1).

### Participation and Recruitment

#### Inclusion

To participate in this study, one must be an undergraduate student at East Carolina University majoring in a prehealth field. Prehealth majors include but are not limited to biology, chemistry, psychology, public health, physics, kinesiology, and recreation therapy.

#### Exclusion

Undergraduate students who are not in a prehealth major (eg, business, computer science, English, or geology) and graduate students will be excluded.

#### Recruitment

Participants for this study will be recruited from undergraduate prehealth programs. The university offers multiple programs, including but not limited to nursing, physician assistant, physical therapy, occupational therapy, speech therapy, recreational therapy, and audiology programs. The university is known for its large population of prehealth students.

The primary recruitment form will be distributed via GroupMe and social media messaging. We will verbally announce the same message to Buff in Scrubs organization members at meetings. Emails will be sent to students via the university honors program and the preprofessional advising center’s weekly newsletter. Flyers will be posted on campus in the following locations: dormitories, the public health building, and the library.

### Pathway to Health Professions Intervention

Pathway to Health Professions consists of 6 sessions; each session was developed based on literature focusing on IPE to improve undergraduate student self-efficacy (Table 1). At the conclusion of each IPE session, the participants will be asked to complete a retrospective quantitative survey.

**Table 1.** Pathway to Health Professions sessions.

Sessions	Session description	Self-efficacy skills	References
Session 1: Mentor Match Up	Included participants will be paired up with students currently in graduate health programs. During this matchup, undergraduate students will ask graduate students questions and advice about their specific programs.	Self-esteem Being informed about tasks to reach goals Confidence in ability to network Confidence in written and verbal communication Confidence in ability to perform tasks and activities required to achieve career goals	[23,24]
Session 2: Rounds Revealed	During this session, students will be provided with literature and videos that outline what it would look like to shadow a physician. Students will also have an opportunity to talk about their own experiences while shadowing and what they learned.	Confidence in ability to network Confidence in cold communication with health care providers Ability to ask questions and learn from health care providers	[2,25]
Session 3: Medical Mock Trials	During this session, students will pair up and portray mock patient-health care provider relationships to gain an understanding of the need for patient education. Students will portray diverse types of providers and work together on a case study, mirroring real-life patient care teams.	Consideration of whether the chosen health career is suitable for the student	[26,27]
Session 4: Equity in Action: Health Challenges and Solutions	During this session, a guest speaker will convey the importance of health equity and how providers can work together to address issues patients face. The speaker will address how different populations face different issues and how, as future providers, the students’ job is to reduce those differences.	Confidence in ability to work effectively with medically underserved communities Understanding social, economic, historical, political, psychosocial, and cultural factors that influence the health of underserved populations Familiarity with community resources available to assist members of health care teams that treat underserved populations Confidence in ability to communicate with and assist people with different needs	[13,28]
Session 5: Pathway Prep: Application and Shadowing Tips/Graduate Applications Walkthrough	During this session, similarities and differences among the most popular graduate program application processes (MD, PT^a^, PA^b^, dentistry, and nursing) for current Buff in Scrubs students will be discussed and reviewed. A slideshow presentation detailing application requirements and recommendations will be shown. For shadowing, students will be provided with guidance on how to contact physicians and a sample email template. Students can begin the application process during the session and ask questions. The 3 most popular processes will be determined based on the members’ interests.	Preparedness to present oneself during an interview Confidence in ability to perform all the tasks and activities required to achieve health science career goals	[29,30]
Session 6: Health Roles Unveiled: Occupation Walkthroughs or Profession Profiles: Interprofessional Skits and Talks	During this session, students will learn about different professions and what a day in the life looks like. This will encourage students to learn more about different occupations. Additionally, it will allow participants to be aware of what their counterparts in other professions do when treating patients.	Being informed about what needs to be done to reach the goal of becoming a health science professional Ability to see oneself as capable of achieving and practicing their chosen health science career	[30,31]

a PT: physical therapist.

b PA: physician assistant.

The 6 sessions (Table 1) will be completed over 3 months, with two 90-minute or 1-hour sessions per month. Sessions will address common concerns or issues that prehealth students experience (Multimedia Appendix 1). Each session has been developed through a literature review and is characterized by various objectives that have been shown to benefit prehealth students in multiple studies.

### Implementation of the Intervention

A detailed description of each session is provided in Multimedia Appendix 1. This description includes the purpose, name of the session, location of the session, session description, materials needed, the goals and objectives, the facilitation process, variations to the session, and references. At the conclusion of each session, a survey will be administered. Participants will scan a QR code and complete the survey on a personal device. The same instructions will be given to participants by each session facilitator to ensure consistent delivery of the survey. Participants will be told that survey participation is optional and anonymous to reduce expectation and confirmation bias.

### Outcomes

#### Measures and Outcomes

Six surveys (Multimedia Appendix 2) were developed comprising a Likert scale using questions from academically sourced journals. This will allow the survey to be reputable and valid. Surveys and questionnaires that assess self-efficacy were used as sources. Some questions’ wording was altered to fit the Pathway to Health Professions program language. These alterations were small and would not affect the validity of the questions.

At a student’s first session in the program, they complete a brief demographic questionnaire that includes current year of study, age, gender, race, ethnicity, and aspired profession. Participants also complete a brief survey at the end of each session that includes demographics, a repeated primary outcome measure, session-specific items, and open-ended feedback.

The primary outcome is self-efficacy, assessed using the same 6 items at each session (refer to Multimedia Appendix 1 for the exact wording). Four items use Likert-type agreement response options (“strongly disagree” to “strongly agree”), and 2 items use a confidence response option (“not confident” to “completely confident”). Each session also includes topic-specific questions unique to that session, all assessed using a Likert-type agreement scale (“strongly disagree” to “strongly agree”). The number of session-specific items varies by session: session 1 (1 item), session 2 (2 items), session 3 (1 item), session 4 (4 items), session 5 (2 items), and session 6 (1 item). In addition, participants respond to a global program satisfaction item (“program recommendation”) rated from “not at all recommend” to “highly recommend” and an open-ended qualitative item (“Please share your feedback”). Open-ended responses are used to capture qualitative suggestions for program improvement. Surveys are administered using Qualtrics (Qualtrics International Inc) at the end of each session. Participants will be informed that completing the surveys is optional.

#### Survey Matching Procedures

We will follow a previously established method that will use facts about a person and generate a numbered code to match them to their responses [32]. For our surveys, we will use the number of older siblings of the participants, the name of their first pet, the color of their backpack, and the last 2 digits of their phone number. This will allow us to match the survey data anonymously. Furthermore, matching will allow us to assess change over time in the participant.

### Analytic Methods

A Wilcoxon signed-rank test will be used to assess changes in responses over time for Likert-scale data. The Likert-scale items, ranging from, for example, “strongly disagree” (1) to “strongly agree” (5), will be treated as ordinal data, and changes will be examined retrospectively during each session.

Given the ordinal nature of Likert-scale data, nonparametric tests will be used over parametric methods that assume normality. Specifically, the Friedman test will be used to assess the overall differences in median scores across multiple time points. Post hoc pairwise comparisons with Bonferroni correction will be performed if the Friedman test indicates a statistically significant result. All analyses will be conducted using SPSS (version 28; IBM Corp).

### Ethical Considerations

This study has been approved by the East Carolina University and Medical Center Institutional Review Board (UMCIRB 24-001921).

Informed consent was obtained from all participants prior to data collection. Participants were provided with detailed information about the study purpose, procedures, risks, and benefits and were informed that their participation was voluntary and that they could withdraw at any time without penalty. Participants were not paid.

To support anonymous tracking of responses across multiple sessions, participants were asked to generate responses to a set of anonymous identification questions (see Survey Matching Procedures section). These identifiers were designed to protect participant confidentiality while allowing for linkage of responses over time. No personally identifiable information was collected, and all data were stored securely in accordance with institutional review board guidelines.

## Results

Data were collected from January 2025 to April 2025. As of June 2025, 9 individuals were recruited. Data analysis is currently underway. Results are expected to be published in January 2026.

## Discussion

### Expected Findings

Pathway to Health Professions is designed to strengthen prehealth undergraduates’ self-efficacy for pursuing graduate health profession training. We anticipate that students who participate in all 6 IPE-based sessions over the 3-month period will report meaningful improvements in perceived preparedness for graduate applications and interviews, confidence in networking and communicating with health professionals, clarity regarding career fit, and confidence in working with medically underserved communities. We further hypothesize that repeated exposure to interprofessional content and role models across sessions will reinforce students’ sense of belonging in health professions and their belief that they can successfully navigate the pathway to graduate training.

This protocol offers several contributions. First, it provides a replicable, session-by-session structure for an IPE-based prehealth preparation program that can be adopted or adapted by institutions seeking to strengthen their health profession pipeline. Second, the curriculum is explicitly mapped to specific self-efficacy domains (eg, confidence in networking, communicating with health care providers, navigating graduate program applications, and working with underserved communities), allowing future evaluation to examine which components are most strongly associated with gains in different domains. Third, the program brings together multiple types of experiential learning, mentoring, reflective discussion, simulation, and career exploration within an interprofessional framework, which may better mirror the collaborative nature of contemporary health care.

A further strength is the program’s emphasis on equity. The inclusion of a dedicated health equity session and the repeated attention to medically underserved populations across activities are intended to reinforce students’ sense of responsibility for reducing health disparities and align their career aspirations with the needs of marginalized communities. Finally, the program is designed to be feasible within a typical academic semester (six 90-minute sessions over 3 months), which may facilitate adoption at institutions with limited curricular flexibility.

### Future Directions and Dissemination

Following implementation and evaluation, we plan to refine the program based on participant feedback, outcome data, and feasibility considerations. Future work could include longer-term follow-up to assess maintenance of self-efficacy gains and downstream outcomes such as graduate program applications, acceptances, and persistence in health profession training. Adaptations for different institution types (eg, community colleges and minority-serving institutions) and delivery formats (hybrid or fully online) will also be important to enhance scalability. In addition, subgroup analyses (eg, by first-generation status or underrepresented racial or ethnic groups) may help determine whether the program differentially benefits students who have historically faced barriers to entering health professions.

Dissemination of the Pathway to Health Professions program will occur through multiple channels, including open access publication of the curriculum, sharing of session materials with campus partners, and presentations. If future evaluations demonstrate positive effects on student self-efficacy and graduate school readiness, this protocol could serve as a model for institutions seeking structured, equity-oriented IPE experiences to strengthen the health profession workforce pipeline.

### Limitations

This protocol has several limitations, the first of which is the surveys. The surveys themselves may be difficult for students to complete. For example, we may have students who attend a certain session but not others, so we will not have a full dataset including all 6 sessions for each student. The Likert scale itself has limitations. Likert scales present an inherent bias that can affect how respondents answer questions. Furthermore, the scale does not offer many options and limits respondents to answers they may disagree with [33]. The retrospective nature of the survey introduces potential recall bias and may affect the accuracy of the results. We will attempt to mitigate recall bias by conducting the surveys immediately following each session.

### Conclusions

This protocol paper outlines Pathway to Health Professions, a prehealth preparation IPE program. Future testing of the use of this program is outlined. We hypothesize that the results from the future testing of this program will show an improvement in undergraduate prehealth students’ self-efficacy related to graduate school readiness.

## Supplementary material

10.2196/74394 Multimedia Appendix 1 Session descriptions.

10.2196/74394 Multimedia Appendix 2 Session surveys.

## Abbreviations

IPE: interprofessional education
