# Supplementary material for: Undergraduate Interprofessional Education: Protocol for a Mixed Methods Study
Source: JMIR Res Protoc. 2026 Apr 8;15:e74394. doi: 10.2196/74394 (PMC13060738; doi:10.2196/74394)
Supplement: Multimedia Appendix 1 [file resprot-v15-e74394-s001.pdf]

Session 1- Mentor Match Up

Purpose:
Match pre-health undergraduate students with students currently in graduate health programs.

Name of Session:
Mentor Match Up

Location of Session:
TBD

Session Description:
Participants will be paired with students currently in graduate health programs. During this match-up, undergraduate students will have the opportunity to ask graduate students questions about the program and ask for advice.

Material Needed:

- Name Tag Stickers, Markers, List of questions for the mentee to ask mentor "title", questions for speed dating, timer, buzzer

Goals/Objectives of Session:
Goal: Undergraduate students will have a graduate student mentor who matches their intended career and interests by the end of the session.
Objectives:
1). Prior to the session, undergraduate students will choose 3 questions to ask their chosen graduate student mentor from the Mentee Questions" (see Appendix A)."
2). By the end of the session, undergraduate students will have asked 3 questions to their chosen graduate student mentor.
3). By the end of the session, undergraduate students will have exchanged contact information (email, Instagram, phone number) with their chosen graduate student mentor.

Facilitation Process:

- 5 minutes- Welcome attendees and have them put their name and program on name tag stickers
- 5 minutes - Introduce the goal and objectives along with the agenda of the session. Answer any questions.
- 10 minutes- Split into groups based on intended careers and introduce mentors for each.
- 40 minutes- "Speed dating," where students and mentors rotate around and meet each other. Participants will physically be in different areas based on their careers to prevent cross-career matching. Students and mentors are seated at desks across from each other. They will begin by utilizing 3 of the questions from Appendix A. Every 2 minutes, a buzzer is rung to signal changing seats. Students will rotate clockwise to their next mentor. This will repeat until all mentees have had a chance to speak with all mentors.
- 10 minutes- Assign mentors and students based on their rank lists.
- 5 minutes – Mentor/Mentee will exchange contact information.

- 5 minutes- Wrap up. Answer any questions.
- 10 minutes – Retrospective Survey.

Variations to Session:
If the speed dating format is not working, it can be changed to simply assigning mentors and students together. If there are not enough mentors, then there will be multiple mentees per group.

References

Brown, B. K., & Hanson, S. H. (2003). Development of a student mentoring program. American Journal of Pharmaceutical Education, 67(1), 947-953. Retrieved from https://www.proquest.com/scholarly-journals/development-student-mentoring-program/docview/211190664/se-2

Nimmons, D., Giny, S., & Rosenthal, J. (2019). Medical student mentoring programs: current insights. *Advances in Medical Education and Practice*, *10*, 113–123. https://doi.org/10.2147/AMEP.S154974

Session 2:

Purpose:
Show students what guidelines to follow and best practices when shadowing a physician or healthcare provider.

Name of Session: Rounds Revealed

Location of Session:
TBD

Session Description:
During this session, students will be provided with literature and videos that entail what it would look like to be shadowing various healthcare professions. Students will also have an opportunity to talk about their own experiences while shadowing, and what they were able to learn.

Material Needed:
- Papers for students to take notes
- Papers will have some guiding questions (Appendix B) on them

Goals/Objectives of Session:
Goal: During this session, students will gain a clear understanding of shadowing healthcare professionals.
Objectives:
1. During this session, students will be expected to engage with the provided educational materials (see Appendix C) by taking notes.
2. During the session, students will engage in peer discussions by providing their thoughts and opinions.
3. During the discussion portion of the session, students will list three insights from the educational materials.
4. At the end of the session, students will share a personal story based on a past shadow experience with a healthcare provider in their small group.
5. At the end of the session, students will report whether the information they received would have helped them in their past shadowing experience.

Facilitation Process:
- 5 minutes- Welcome attendees and have them put their name and program on name tag stickers
- 5 minutes - Introduce the goal and objectives along with the agenda of the session. Answer any questions.
- 5 minutes- Students group themselves according to profession; if there is a lone person, they will be added to another group closely related to their profession.
- 20 minutes- students will read through and watch videos about their profession and discuss what they read/watched (Appendix C).

- 20 minutes- Students can share personal stories about past shadowing experiences and what they have learned from the session.
- 5 minutes - Wrap-up
- 10 minutes – Retrospective Survey

Variations to Session:
If there is an issue with this session, students instead can simply provide short personal shadowing stories on what they have noticed during shadowing and what they would tell incoming students. They could also possibly develop an infographic with helpful tips. If a student has not had past shadowing experience, they can talk about how they plan to use the things they have learned in the future.

References:

Hamilton, D., Taylor, C., & Maben, J. (2023). How Does a Group Reflection Intervention (Schwartz Rounds) Work within Healthcare Undergraduate Settings? A Realist Review. *Perspectives on medical education*, *12*(1), 550–564. https://doi.org/10.5334/pme.930

Walton, V., Hogden, A., Long, J. C., Johnson, J. K., & Greenfield, D. (2019). How Do Interprofessional Healthcare Teams Perceive the Benefits and Challenges of Interdisciplinary Ward Rounds. *Journal of Multidisciplinary Healthcare*, *12*, 1023–1032. https://doi.org/10.2147/JMDH.S226330

Session 3:

Purpose:
Assist students in developing effective communication and patient education skills by practicing mock patient-provider interactions and fostering teamwork skills by collaborating on a case study that mirrors real-life patient care scenarios.

Name of Session:
Medical Mock Trials

Location of Session:
TBD

Session Description:
During this session, students will pair up and portray mock patient-provider relationships. They will have the chance to portray diverse types of providers and work together on a case study, mirroring real-life patient care teams.

Material Needed:
- Printed case studies
- Role cards (for various provider roles: doctor, nurse, pharmacist, social worker, etc.)
- Writing materials (notebooks, pens)
- Patient information sheets
- Whiteboard and markers
- Timer or stopwatch
- Chairs and tables arranged for team discussions

Goals/Objectives of Session:

Goal: At the end of this session, students will develop teamwork skills and an understanding of interdisciplinary care through a collaborative case study.

Objectives:

1. During the session, students will assume different healthcare roles related to their intended careers and work together on a case study.
2. At the end of the session, students will be able to present their case study solutions and receive feedback on their teamwork and approach.

Facilitation Process:
- 10 minutes- Introduction- Facilitator (club executive members) introduces the session objectives and the importance of patient-provider communication and interdisciplinary collaboration. Brief overview of the mock trial format and the roles to be assumed.
- 10 minutes- Role Assignment and Preparation- Students are paired up and assigned roles (patient or various types of providers). Role card sheets are distributed. Students will sit

with others of the same career. They will count off “1 2 3 4 etc.” for how many groups will be made. This will result in a few students from each pre-health career in each group.

- 50 minutes- Case Study Group Work- Students form small, multidisciplinary teams to work on a provided case study which is provided from (Appendix D). Teams discuss the case, assign roles, and create a plan of action, simulating a real-life patient care team meeting.
- 15 minutes- Group Presentations and Feedback- Each group presents their case study solution and approach. Facilitator and peers provide constructive feedback on teamwork, communication, and problem-solving.
- 5 minutes- Debrief and Reflection- Facilitator leads a group discussion reflecting on key takeaways, challenges, and lessons learned from the mock trials and case study exercise.
- 10 minutes – Retrospective Survey

Variations to Session:

- Role Rotations: If time allows, students can rotate roles more than once to experience different perspectives within the healthcare team.
- Advanced Scenarios: For more experienced students, more complex case studies can be introduced, incorporating ethical dilemmas or emergency situations.
- Telehealth Adaptation: If conducted online, breakout rooms can be used for mock interactions, and virtual tools like shared documents can facilitate case study discussions.

References:

Flanagan, O. L., & Cummings, K. M. (2023). Standardized Patients in Medical Education: A Review of the Literature. *Cureus*, *15*(7), e42027. https://doi.org/10.7759/cureus.42027

Song, C., & Jang, A. (2023). Mock trial as a simulation strategy allowing undergraduate nursing students to experience evidence-based practice: A scoping-review. PLOS ONE, 18(8). https://doi.org/10.1371/journal.pone.0289789

Session 4:

Purpose:
Educate students on the importance of health equity, the challenges faced by different populations, and the role of healthcare providers in reducing health disparities.

Name of Session:
Equity in Action: Health Challenges and Solutions

Location of Session:
TBD

Session Description:
During this session, a guest speaker will discuss the importance of health equity and how providers can work together to address patients' issues. The speaker will also address how different populations face different issues and how our job as future providers is to reduce those differences.

Material Needed:
- Water and refreshments for the speaker and participants
- Students will bring notepads and writing utensils

Goals/Objectives of Session:

Goal: At the end of the session, students should be able to understand the importance of health equity and identify actionable steps to address health disparities.

Objectives:

1. Students will be able to define health equity by the end of the session.
2. During the session students will ask the guest speaker a minimum of 1 question on health equity.
3. Students will identify two specific actions they can take to promote health equality as future healthcare providers.
4. At the end of the session, students will verbally report 1 action that they can promote and provide a specific example on how they will promote this action.

Facilitation Process:
- 5-minute Introduction- The Facilitator welcomes participants, introduces the guest speaker, and provides an overview of the session's objectives.
- 30-Guest Speaker The guest speaker discusses the importance of health equity, the challenges faced by different populations, and the role of healthcare providers in addressing these issues.
- 15 minutes- Speaker provides specific examples and strategies for reducing health disparities.

- 15 minute-Q&A Session-This is an Open floor for students to ask questions, share thoughts, or discuss challenges related to health equity. The facilitator moderates the Q&A, ensuring a diverse range of questions are addressed.
- 10 minutes - Reflection and Wrap-Up- Students are asked to write a brief reflection on what they learned and two specific actions they plan to take to promote health equity in their future practice. The facilitator collects reflections and provides closing remarks, thanking the guest speaker and participants.
- 10 minutes- Retrospective Survey

Variations to Session:

- Panel Discussion: Instead of a single guest speaker, a panel of experts representing various healthcare fields can be invited to provide multiple perspectives on health equity.
- Interactive Workshop: If time allows, an additional interactive segment can be included where students work in small groups to brainstorm solutions to specific health equity challenges presented by the speaker.

References:

Cooper LA, Purnell TS, Showell NN, et al. Progress on Major Public Health Challenges: The Importance of Equity. Public Health Reports®. 2018;133(1_suppl):15S-19S. doi:10.1177/0033354918795164

Maldonado, M. E., Fried, E. D., DuBose, T. D., Nelson, C., & Breida, M. (2014). The role that graduate medical education must play in ensuring health equity and eliminating health care disparities. *Annals of the American Thoracic Society*, *11*(4), 603–607. https://doi.org/10.1513/annalsats.201402-068ps

Session 5:

Purpose: The purpose of this session is to introduce and walk students through graduate applications.

Name of Session: *What does it mean to be an MD, DO, PA, PT, OT, RT, Dentist, Speech Therapist, Social Worker, or Nurse?*

Location of Session: TBD

Session Description: During this session, we will go over the similarities and differences of the three most popular application processes (MD*, DO, PA, PT, OT, RT, Dentist, Speech Therapist, Social Worker, or Nurse*). This will give students a head starts on the process and give them an opportunity to ask questions.

Material Needed:
- Laptop and projector
- Kahoot with questions that ask relevant questions to the applications
- Students will need notetaking devices (paper, iPad, etc.)

Goals/Objectives of Session: (Write goals and objectives for the session – use SMART criteria).
Goal: At the end of this session students should be able to identify the similarities and differences in different graduate application processes.
1). By the end of this session, we hope students can describe the application process for certain graduate programs and draw similarities and differences between them.

2). At the end of the session, a quick Kahoot game will be played where students must match the process with the correct graduate program.

3). At the end of the session, students will know what the application process looks like, what's expected, and general knowledge, which will be demonstrated by completing session activities.

Facilitation Process:
- 5 minutes- Welcome attendees and have them put their name and program on name tag stickers
- 5 minutes - Introduce the goal and objectives along with the agenda of the session. Answer any questions.
- 40 minutes- Go through application processes, what's required, different tests (MCAT, GRE CASPER, etc.)
- 15 minute- Kahoot review (Appendix E)
- 5 minute- answer any questions students may have
- 10 minutes – Retrospective Survey

Variations to Session:

- Application Panel: Bring in current students to talk about how they completed the application process, this would lead to reduced graduate programs, maybe only discussing 2-3

References:

Castillo, E. (2020). Graduate school application accessibility for international students (Order No. 27962055). Available from ProQuest Dissertations & Theses Global. (2423836940). Retrieved from https://www.proquest.com/dissertations-theses/graduate-school-application-accessibility/docview/2423836940/se-2

Estien, C., Chapman , M., Schell, C. J., Lowy, N., & Gerson, J. R. (2022). Demystifying the Graduate School Application Process. doi: https://doi.org/10.1002/bes2.2029

Session 6:

Purpose: Give students the opportunity to see what a future in career in certain health fields looks like

Name of Session: *Health Roles Unveiled: Occupation Walkthroughs or Profession Profiles: Interprofessional Skits and Talks*

Location of Session: TBD

Session Description: During this session students will be able to learn more about different professions and what 'Day in the Life of...' looks like. This will hopefully encourage students to want to learn more about different occupations and be able to keep in mind what their counterparts do when treating patients.

Material Needed:

- Videos
- Literature
- Laptops, notetaking devices

Goals/Objectives of Session:
Goals: At the end of this session students will have a better understanding of various health professionals' lives.

1. At the end of this session students will be able to identify what it looks like to work in a respective field and be able to engage in discussions.
2. After this session students will be confident in being able to identify the roles that different careers play in the health system and be able to connect the different toles together and how they affect each other.

Facilitation Process:

- 5 minutes- Welcome attendees and have them put their name and program on name tag stickers
- 5 minutes - Introduce the goal and objectives along with the agenda of the session. Answer any questions.
- 40 minutes- Introduce videos to show what different providers do during their workday. Engage with the group to talk about the difference between their work looks like.
- 20 minutes- Discuss what was presented via small groups.
- 10 minutes – Retrospective Survey

Variations to Session:

- Give students the time to pick a group to research and put together a fun design board and present what they found

References:

Rhonda L Beggs, Danny K Sidwell, Megan E Rattray, Georgina E Neville, Peta-Anne P Zimmerman, Undergraduate nursing students' perceptions of the graduate application process: A qualitative exploration, Collegian, Volume 29, Issue 2, 2022, Pages 213-219, ISSN 1322-7696, https://doi.org/10.1016/j.colegn.2021.08.006.(https://www.sciencedirect.com/science/article/pii/S1322769621001037)

Estien, C., Chapman , M., Schell, C. J., Lowy, N., & Gerson, J. R. (2022). Demystifying the Graduate School Application Process. doi: https://doi.org/10.1002/bes2.2029

APPENDIX A

General Questions for All Health-Related Graduate Programs

What does a typical day look like for you?

How do you manage your time between classes, studying, and personal life?

What are the biggest challenges you’ve faced so far?

How do you handle stress and maintain your mental health?

What resources are available for academic and personal support?

How do you balance clinical practice with coursework?

What do you wish you had known before starting your program?

How do you prepare for exams and practical assessments?

What opportunities are there for research or specialization?

How do you finance your education and manage expenses?

Appendix B

Questions to ask for videos and articles

General Comprehension Questions

1. What was the main topic of the article/video?
2. Can you summarize the key points discussed?
3. What new information did you learn?

Critical Thinking Questions

1. Do you agree or disagree with the information presented? Why?
2. How does this information relate to what you already know about the topic?
3. What are the potential implications of this information for your own past shadowing experiences?

Application Questions

1. How can you apply the information from the article/video to future shadowing experiences?
2. What changes might you consider making based on what you learned?
3. Can you think of any real-life examples that illustrate the points made in the article/video?

Discussion Questions

1. What questions do you still have after reading/watching?
2. How did the article/video make you feel about the topic?
3. What was the most surprising or interesting fact you learned?

Appendix C

Articles and Videos for Interdisciplinary Rounding

Interdisciplinary Rounds – Christiana Care

https://youtu.be/C31k-CU6Bqg?si=eQcGj8MvvdmLRhwc

Hospitalist Rounds – Diabetic Neuropathy, Stepped on Nail

https://youtu.be/MSU_ZSZcb_4?si=iaOaQW6NE_s8AOep

Interdisciplinary Simulation Scenario – Start at 0:35

https://youtu.be/rY9yFs6bxuE?si=AXG66fvtrJ1C1Nh3

Medical Ward Rounds – Potential Asthma

https://youtu.be/FnOP3PA8zCI?si=ESF43SBHutTA9BUA

How to do Interdisciplinary Rounds PDF

https://www.saskhealthquality.ca/wp-content/uploads/2021/08/Interdisciplinary-Rounding-Module-July-2017.pdf

Appendix D

Case 1: Elderly Patient with Multiple Chronic Conditions

Patient: 78-year-old female **History:** Hypertension, Type 2 Diabetes, Chronic Kidney Disease, Osteoarthritis

Current Issue: The patient presents with worsening kidney function, uncontrolled blood sugar levels, and increased joint pain.

Interprofessional Focus:

- **Doctor:** Adjust medications for kidney function and diabetes.
- **Nurse:** Monitor vital signs, educate the patient on lifestyle changes.
- **Dentist:** Address any oral health issues that may affect overall health.
- **Physician Assistant:** Assist with routine check-ups and follow-up care.
- **Physical Therapist:** Develop a low-impact exercise plan to manage osteoarthritis.
- **Social Worker:** Assess home environment and provide resources for support services.
- **Respiratory Therapist:** Monitor respiratory status, especially if there are any complications from chronic conditions.
- **Recreational Therapist:** Engage the patient in activities to improve quality of life and mental health.

Case 2: Young Adult with Mental Health and Substance Abuse Issues

Patient: 25-year-old male **History:** Depression, Anxiety, Alcohol Use Disorder

Current Issue: The patient has been experiencing severe anxiety attacks and has recently relapsed into heavy drinking.

Interprofessional Focus:

- **Doctor (Psychiatrist):** Adjust psychiatric medications and provide therapy.
- **Nurse:** Monitor mental health status and provide support.
- **Dentist:** Address any dental issues related to substance abuse.
- **Physician Assistant:** Assist with mental health assessments and follow-up care.
- **Physical Therapist:** Develop a physical activity plan to improve mental health.
- **Social Worker:** Connect the patient with substance abuse programs and support groups.
- **Respiratory Therapist:** Monitor respiratory health, especially if there are complications from substance abuse.

- **Recreational Therapist:** Engage the patient in therapeutic activities to support recovery.

Case 3: Middle-Aged Patient with Post-Surgical Complications

Patient: 50-year-old female **History:** Recent knee replacement surgery, Hypertension

Current Issue: The patient presents with signs of infection at the surgical site and uncontrolled blood pressure.

Interprofessional Focus:

- **Doctor (Surgeon):** Evaluate and treat the surgical site infection.
- **Nurse:** Provide wound care and monitor for signs of infection.
- **Dentist:** Ensure oral health is maintained to prevent any additional infections.
- **Physician Assistant:** Assist with post-surgical follow-up and blood pressure management.
- **Physical Therapist:** Modify rehabilitation plan to accommodate infection.
- **Social Worker:** Assist with post-surgical care resources and support.
- **Respiratory Therapist:** Monitor respiratory status, especially if the patient is bedridden.
- **Recreational Therapist:** Provide activities to aid in recovery and improve mental well-being.

Case 4: Pediatric Patient with Asthma and Allergies

Patient: 8-year-old male **History:** Asthma, Severe Allergies

Current Issue: The patient has had multiple asthma attacks triggered by seasonal allergies.

Interprofessional Focus:

- **Doctor (Pediatrician):** Adjust asthma and allergy medications.
- **Nurse:** Educate the family on asthma management and trigger avoidance.
- **Dentist:** Monitor oral health, especially if the patient uses inhalers frequently.
- **Physician Assistant:** Assist with routine check-ups and asthma management.
- **Physical Therapist:** Provide breathing exercises and techniques.
- **Social Worker:** Connect the family with community resources for allergy management.
- **Respiratory Therapist:** Provide specialized care for asthma management.
- **Recreational Therapist:** Engage the child in activities that are safe and enjoyable, considering their asthma and allergies.

Case 5: Adolescent with Traumatic Brain Injury

Patient: 16-year-old male **History:** Traumatic Brain Injury (TBI) from a car accident

Current Issue: The patient is experiencing cognitive impairments, motor function difficulties, and emotional instability. **I**

nterprofessional Focus:

- **Doctor (Neurologist):** Manage and monitor neurological status.
- **Nurse:** Provide daily care and monitor for changes in condition.
- **Dentist:** Address any dental trauma from the accident.
- **Physician Assistant:** Assist with routine assessments and follow-up care.
- **Physical Therapist:** Develop a rehabilitation plan to improve motor function.
- **Social Worker:** Provide support to the family and connect them with resources.
- **Respiratory Therapist:** Monitor respiratory function, especially if the patient has difficulty swallowing.
- **Recreational Therapist:** Engage the patient in activities to support cognitive and emotional recovery.

Case 6: Adult with Severe Burn Injuries

Patient: 35-year-old female **History:** Severe burns covering 40% of the body from a house fire

Current Issue: The patient is dealing with pain management, risk of infection, and mobility issues.

Interprofessional Focus:

- **Doctor (Burn Specialist):** Oversee burn treatment and pain management.
- **Nurse:** Provide wound care and monitor for signs of infection.
- **Dentist:** Ensure oral health is maintained, especially if the patient is intubated.
- **Physician Assistant:** Assist with daily care and follow-up assessments.
- **Physical Therapist:** Develop a plan to improve mobility and prevent contractures.
- **Social Worker:** Support the patient and family with resources and counseling.
- **Respiratory Therapist:** Monitor respiratory status, especially if there are inhalation injuries.
- **Recreational Therapist:** Provide activities to improve mental well-being and aid in recovery.

Case 7: Pregnant Woman with Gestational Diabetes

Patient: 28-year-old female **History:** Gestational Diabetes, 32 weeks pregnant

Current Issue: The patient is experiencing difficulty managing blood sugar levels and is at risk for preterm labor.

Interprofessional Focus:

- **Doctor (Obstetrician):** Manage pregnancy and gestational diabetes.
- **Nurse:** Monitor maternal and fetal health, provide education on diabetes management.
- **Dentist:** Ensure oral health is maintained, as pregnancy can affect dental health.
- **Physician Assistant:** Assist with routine prenatal care and diabetes management.
- **Physical Therapist:** Develop a safe exercise plan to help manage blood sugar levels.
- **Social Worker:** Provide resources and support for managing pregnancy and diabetes.
- **Respiratory Therapist:** Monitor respiratory status, especially if the patient has any complications.
- **Recreational Therapist:** Engage the patient in stress-reducing activities to support overall health.

Case 8: Elderly Patient with Dementia and Pneumonia

Patient: 82-year-old male **History:** Dementia, Recurrent Pneumonia

Current Issue: The patient is currently hospitalized with pneumonia and is experiencing confusion and agitation.

Interprofessional Focus:

- **Doctor (Geriatrician):** Manage dementia and pneumonia treatment.
- **Nurse:** Provide daily care, monitor vital signs, and manage agitation.
- **Dentist:** Address any oral health issues that may contribute to pneumonia.
- **Physician Assistant:** Assist with routine assessments and follow-up care.
- **Physical Therapist:** Develop a plan to improve mobility and prevent pneumonia recurrence.
- **Social Worker:** Support the patient and family with resources and counseling.
- **Respiratory Therapist:** Provide respiratory care and monitor lung function.
- **Recreational Therapist:** Engage the patient in activities to reduce agitation and improve quality of life.

Example

Multidisciplinary Team Rounds

Dr. Smith (MD): “Good morning, team. Let’s start with Alex Johnson. He had knee surgery three days ago. How is he doing, Nurse Kelly?”

Nurse Kelly: “Alex’s vitals are stable, and his pain is manageable with the prescribed medication. He’s been compliant with his medication schedule.”

Dr. Lee (DO): “I’ve checked his range of motion, and while it’s still limited, he’s making progress. We’ll continue with gentle exercises and osteopathic manipulative treatment to aid his recovery.”

Jamie (PA): “I changed his bandages this morning. The incision site looks clean with no signs of infection. Alex reports that the pain is consistent but manageable.”

Sam (PT): “We’ve started with light exercises to strengthen his knee. Alex is responding well, though he finds some movements challenging. We’ll gradually increase the intensity as he improves.”

Taylor (OT): “Alex mentioned difficulty with stairs at home. We’re working on techniques to help him manage daily activities more easily. I’ll provide him with some adaptive equipment if needed.”

Chris (RT): “I’ve checked his respiratory status, and there are no issues. His breathing is normal, and there are no signs of respiratory complications post-surgery.”

Dr. Patel (Dentist): “No dental concerns at the moment. I’ve advised Alex to maintain his oral hygiene to prevent any issues during his recovery.”

Jordan (Speech Therapist): “No speech or swallowing issues reported. I’ll remain available if any concerns arise.”

Morgan (Social Worker): “Alex is coping well at home with the support of his family. I’ve provided information on additional resources if he needs further assistance.”

Dr. Smith (MD): “Excellent. It sounds like Alex is on track for a smooth recovery. Let’s continue with the current plan and reassess in a few days. Any other concerns or suggestions?”

Team: “No additional concerns at this time.”

APPENDIX E

Question 1

Q: What is a common requirement for both MD and DO medical school applications?

- A) GRE
- B) MCAT
- C) LSAT
- D) GMAT

Answer: B) MCAT

Question 2

Q: Which of the following professions typically requires the completion of a residency program after graduation?

- A) Physical Therapist (PT)
- B) Physician Assistant (PA)
- C) Doctor of Medicine (MD)
- D) Social Worker

Answer: C) Doctor of Medicine (MD)

Question 3

Q: Which healthcare profession requires passing the National Physical Therapy Examination (NPTE) for licensure?

- A) Occupational Therapist (OT)
- B) Physical Therapist (PT)
- C) Respiratory Therapist (RT)
- D) Speech Therapist

Answer: B) Physical Therapist (PT)

Question 4

Q: What is a common application requirement for both PA (Physician Assistant) and Nurse Practitioner (NP) programs?

- A) Clinical experience
- B) GRE scores

- C) MCAT scores
- D) Dental Admission Test (DAT)

Answer: A) Clinical experience

Question 5

Q: Which profession typically requires the Dental Admission Test (DAT) as part of the application process?

- A) Dentist
- B) Speech Therapist
- C) Social Worker
- D) Nurse

Answer: A) Dentist

Question 6

Q: What is a similarity between the application processes for MD and PA programs?

- A) Both require a residency
- B) Both require clinical experience
- C) Both require the GRE
- D) Both require a bachelor's degree

Answer: D) Both require a bachelor's degree

Question7

Q: Which profession's application process often includes a focus on manual therapy techniques?

- A) Physical Therapist (PT)
- B) Occupational Therapist (OT)
- C) Respiratory Therapist (RT)
- D) Doctor of Osteopathic Medicine (DO)

Answer: D) Doctor of Osteopathic Medicine (DO)

Question 8

Q: Which of the following professions typically requires a master's degree for entry-level practice?

- A) Social Worker
- B) Physical Therapist (PT)

- C) Speech Therapist
- D) All of the above

Answer: D) All of the above

Question 9

Q: What is a key difference in the application process for MD and DO programs compared to PA programs?

- A) Requirement of a bachelor's degree
- B) Taking the MCAT
- C) Clinical experience
- D) GRE scores

Answer: B) Taking the MCAT

Q: Which profession requires the completion of the Centralized Application Service for Physician Assistants (CASPA)?

- A) MD
- B) DO
- C) PA
- D) PT

Answer: C) PA

Question 10

Q: What is a common requirement for both PT (Physical Therapist) and OT (Occupational Therapist) programs?

- A) GRE scores
- B) MCAT scores
- C) Clinical observation hours
- D) Dental Admission Test (DAT)

Answer: C) Clinical observation hours

Question 11

Q: Which profession's application process typically includes the Test of Essential Academic Skills (TEAS)?

- A) Nurse
- B) Dentist

- C) Speech Therapist
- D) Social Worker

Answer: A) Nurse

Question 12

Q: What is a unique aspect of the application process for Speech Therapists compared to other healthcare professions?

- A) Requirement of a bachelor's degree
- B) GRE scores
- C) Clinical fellowship year
- D) MCAT scores

Answer: C) Clinical fellowship year

Question 13

Q: Which profession requires passing the National Board for Respiratory Care (NBRC) exams for licensure?

- A) Physical Therapist (PT)
- B) Respiratory Therapist (RT)
- C) Occupational Therapist (OT)
- D) Social Worker

Answer: B) Respiratory Therapist (RT)

Question 14

Q: Which healthcare profession typically requires the completion of the American Dental Education Association's Associated American Dental Schools Application Service (ADEA AADSAS)?

- A) Dentist
- B) Nurse
- C) Physician Assistant (PA)
- D) Physical Therapist (PT)

Answer: A) Dentist

Question 15

Q: What is a common requirement for both MD and Nurse Practitioner (NP) programs?

- A) GRE scores

- B) Clinical experience
- C) MCAT scores
- D) Dental Admission Test (DAT)

Answer: B) Clinical experience

Question 16

Q: Which profession's application process often includes a focus on psychosocial assessments and interventions?

- A) Physical Therapist (PT)
- B) Social Worker
- C) Respiratory Therapist (RT)
- D) Dentist

Answer: B) Social Worker

Question 17

Q: What is a key difference in the application process for PT (Physical Therapist) programs compared to RT (Respiratory Therapist) programs?

- A) Requirement of a bachelor's degree
- B) Clinical observation hours
- C) GRE scores
- D) MCAT scores

Answer: C) GRE scores

Question 18

Q: Which profession typically requires the completion of the Centralized Application Service for Nursing Programs (NursingCAS)?

- A) Nurse
- B) Dentist
- C) Physician Assistant (PA)
- D) Speech Therapist

Answer: A) Nurse
