# Supplementary material for: Undergraduate Interprofessional Education: Protocol for a Mixed Methods Study
Source: JMIR Res Protoc. 2026 Apr 8;15:e74394. doi: 10.2196/74394 (PMC13060738; doi:10.2196/74394)
Supplement: Multimedia Appendix 2 [file resprot-v15-e74394-s002.pdf]

Intro Paragraph

You are being invited to participate in a research study titled “Pathway to Health Professions” conducted by Hannah Snead and Dhwani Hada, students at East Carolina University in the Department of Health and Human Services. The purpose of this study is to determine if the Buff in Scrubs program sessions (N=6) are effective in improving self-efficiency for undergraduate students in pre-health majors. Our goal is to survey at least 30 undergraduate individuals in pre-health studies at East Carolina University. The survey will take approximately 5 minutes to complete. Your responses will be kept confidential, and no data will be released or used with your identification attached. Your participation in the study is voluntary and you can participate in the session and student organization without participating in the study. You may choose not to answer any or all questions, and you may stop at any time. We will not be able to pay you for the time you volunteer while being in this study. There is no penalty for not taking part in this research study. Please email Hannah Snead at

sneadh22@students.ecu.edu for any research related questions or the University & Medical Center Institutional Review Board (UMCIRB) at 252-744-2914 for questions about your rights as a research participant.

Informed Consent

Title of Research Study: Pathway to Health Professions

Principal Investigator: Hannah Snead
Study Coordinator: Dhwani Hada
Institution, Department or Division: Health Education and Promotion
Address: 3204 Carol Belk Building Greenville, NC 27858
Telephone #: 252-328-2986

Researchers at East Carolina University (ECU) study issues related to society, health problems, environmental problems, behavior problems and the human condition. To do this, we need the help of volunteers who are willing to take part in research.

Why am I being invited to take part in this research?

The purpose of this research is to explore the link between self-efficacy and the increase in graduate school preparation for pre-health undergraduate students. You are being invited to take part in this research because you are an ECU undergraduate majoring in a pre-health major, on the pre-health path. Pre-health majors include the following but are not limited to: (Biology, Chemistry, Psychology, Public Health, Physics, Kinesiology, Recreation Therapy). The decision to take part in this research is yours to make. By doing this research, we hope to learn ‘does self-efficacy explain the relationship between participation in a pre-health preparation program and an undergraduate pre-health student's applying for graduate school programs?

If you volunteer to take part in this research, you will be one of about 100 people to do so.

During this research you will be asked to participate in research-based sessions to increase your confidence for graduate school. You will ask to collaborate with other pre-health professions, guest speakers, discover literature and helpful skills.

Are there reasons I should not take part in this research?

I understand I should not volunteer for this study if I am, under 18 years of age, undergraduate students who are not in a pre-health major (Business, Computer Science, English,

Geology), are not members of the “Buff in Scrubs” club, and/or are graduate students will be excluded from this study.

What other choices do I have if I do not take part in this research?

You can choose not to participate and remain as a club member. You can still participate in the sessions, but we will not collect any data or information from you.

Where is the research going to take place and how long will it last?

The research will be conducted at East Carolina University. You will need to come to Buff in Scrubs club meetings times during the study. The total amount of time you will be asked to volunteer for this study is about nine hours and will take place over the course of 3 months.

What will I be asked to do?

You will be asked to do the following:

- Participate in all six sessions, which will take place over the course of 3 months
- Two sessions per month
- Complete retrospective surveys after each session, to report your thoughts in what the sessions are like

What might I experience if I take part in the research?
We don’t know of any risks (the chance of harm) associated with this research. Any risks that may occur with this research are no more than what you would experience in everyday life. We don't know if you will benefit from taking part in this study. There may not be any personal benefit to you but the information gained by doing this research may help others in the future.

Will I be paid for taking part in this research?
We will not be able to pay you for the time you volunteer while being in this study.

Will it cost me to take part in this research?
It will not cost you any money to be part of the research.

Who will know that I took part in this research and learn personal information about me?
ECU and the people and organizations listed below may know that you took part in this research and may see information about you that is normally kept private. With your permission, these people may use your private information to do this research:
- The University & Medical Center Institutional Review Board (UMCIRB) and its staff have responsibility for overseeing

your welfare during this research and may need to see research records that identify you.

How will you keep the information you collect about me secure? How long will you keep it?

Survey data will be retained for 3 years.

What if I decide I don’t want to continue in this research?

You can stop at any time after it has already started. There will be no consequences if you stop, and you will not be criticized. You will not lose any benefits that you normally receive, such as continuing to be a participant in the Buff in Scrubs club.

Who should I contact if I have questions?

The people conducting this study will be able to answer any questions concerning this research, now or in the future. You may contact the Principal Investigator, Hannah Snead, at sneadh22@students.ecu.edu (Monday – Friday between 8:00 a.m. – 4:00 p.m.

If you have questions about your rights as someone taking part in research, you may call the ECU University and Medical Center Institutional Review Board (UMCIRB) at phone number 252-744-2914 (days). If you would like to report a complaint or concern about this research study, you may call the Director for Human Research Protections, at 252-744-2914.

Is there anything else I should know?

If you do not want to participate in the study, you can still participate in Buff and Scrubs club activities.

This research is overseen by the University and Medical Center Institutional Review Board (UMCIRB) at ECU. Therefore, some of the UMCIRB members or the UMCIRB staff may need to review your research data. Your identity will be evident to those individuals who see this information. However, precautions will be taken to ensure that anyone not authorized to see your identity will not be given that information.

Identifiers might be removed from the identifiable private information, and, after such removal, the information could be used for future research studies or distributed to another investigator for future research studies without additional informed consent from you or your Legally Authorized Representative (LAR). However, there still may

be a chance that someone could discover your identity via the information provided.

I have decided I want to take part in this research. What should I do now?

The person obtaining informed consent will ask you to read the following and if you agree, you should sign this form:

- I have read (or had read to me) all of the above information.
- I have had an opportunity to ask questions about things in this research I did not understand and have received satisfactory answers.
- I know that I can stop taking part in this study at any time.
- By signing this informed consent form, I am not giving up any of my rights.
- I have been given a copy of this consent document, and it is mine to keep.

Do you give your informed consent to participate in this research?

○ No

- Yes

Is this your first session?

Is this the first session of the series that you are attending?

- Yes
- No

Demographics

In which year of your academic program are you currently registered?

- Year 1
- Year 2
- Year 3
- Year 4

What is your age?

What is your gender?

- ◯ Female
- ◯ Male
- ◯ Other
- ◯ Prefer not to answer

What race or races do you consider yourself to be? Please select one or more. Check all that apply.

- ☐ American Indian or Alaska Native
- ☐ Asian
- ☐ Black or African American
- ☐ Native Hawaiian or Pacific Islander
- ☐ White
- ☐ Other
- ☐ Refused

Do you consider yourself to be Hispanic, Latino, or of Spanish origin?

- Yes
- No
- Refused

What is your aspired professional status within the medical field?

- Medical Doctor (M.D.)
- Doctor of Osteopathic Medicine (D.O.)
- Physician Assistant (P.A.)
- Nurse Practitioner (N.P.)
- Dentist (D.D.S. or D.M.D.)
- Physical Therapy (P.T.)
- Occupational Therapy (O.T.)
- Other (Specify):

Matching Questions

What was your first pet's name? If none, write "none".

How many older siblings do you have?

What is the color of your backpack

What are the last 2 digits of your phone number?

Self Efficacy

Please continue filling the form

	Strongly Disagree	Disagree	Not Sure	Agree	Strongly Agree
There is a significant relationship between, self-esteem, self-efficacy, and resource for graduate programs.	○	○	○	○	○

	Strongly Disagree	Disagree	Not Sure	Agree	Strongly Agree
I feel informed about what I need to do to reach my goal of becoming a health/science professional.	○	○	○	○	○
I feel confident in my ability to network.	○	○	○	○	○
I feel confident in my verbal and written communications.	○	○	○	○	○

Please continue filling the form

	Not Confident	Slightly Confident	Somewhat Confident	Confident	Completely Confident
How confident do you feel in your ability to successfully perform all of the tasks and activities required to achieve your health science career goal?	○	○	○	○	○
How confident are you that you will achieve your career goals?	○	○	○	○	○

Which session

Which Session are you taking part in today?

- ○ Session 1 - Speed Dating Mentors
- ○ Session 2 - Shadowing Guidelines
- ○ Session 3 - Practice Patient-Provider Interactions
- ○ Session 4 - Health Equity
- ○ Session 5 - Graduate Applications Walkthrough
- ○ Session 6 - Occupation Walkthroughs

Session 1 - Speed Dating Mentors

Speed Dating Mentors - Session Questions

	Strongly disagree	Disagree a little	Neither agree nor disagree	Agree a little	Strongly agree
I feel confident in my ability to network	○	○	○	○	○

Session 2 - Shadowing Guidelines

Shadowing Guidelines - Session Questions

	Strongly disagree	Disagree a little	Neither agree nor disagree	Agree a little	Strongly agree
Shadowing is useful to determine if healthcare is the right career for me.	○	○	○	○	○
Shadowing is useful to determine which medical specialty is right for me.	○	○	○	○	○

Session 3 – Practice Patient-Provider Interactions

Practice Patient-Provider Interactions - Session Questions

	Strongly Disagree	Disagree a little	Neither agree nor disagree	Agree a little	Strongly agree
I feel the patient-provider interactions walkthrough session provided me an opportunity to seriously consider whether my chosen health career is suitable for me.	○	○	○	○	○

Session 4 - Health Equity

Health Equity - Session Questions

	Strongly Disagree	Disagree	Not Sure	Agree	Strongly agree
I am confident about my ability to work effectively with medically underserved communities.	○	○	○	○	○
I understand the social, economic, historical, political, psychosocial, and cultural factors that influence the health of underserved populations.	○	○	○	○	○
I am familiar with the community resources available to assist members of healthcare teams that treat underserved populations.	○	○	○	○	○
I feel confident in my abilities to communicate with and assist people with different needs.	○	○	○	○	○

Session 5 – Graduate Applications Walkthrough

Graduate Applications Walkthrough - Session Questions

	Strongly Disagree	Disagree	Not Sure	Agree	Strongly agree
I feel prepared on how to present myself during an interview.	○	○	○	○	○

Graduate Applications Walkthrough - Session Questions

	Not confident	Slightly Confident	Somewhat Confident	Confident	Completely Confident
How confident do you feel in your ability to successfully perform all of the tasks and activities required to achieve your health science career goal?	○	○	○	○	○

Session 6 – Occupation Walkthroughs

Occupation Walkthrough - Session Questions

	Strongly disagree	Disagree a little	Neither agree nor disagree	Agree a little	Strongly agree
I feel that this occupation walkthrough class increased my interest in pursuing my chosen career as a job.	○	○	○	○	○

Feedback for session

Please share your feedback for this session.

Wrap Up Questions

Please rank the sessions in order of how much they helped you along your career journey. 1 = most useful, 6 = least useful

Session 1 - Speed Dating Mentors

Session 2 - Shadowing Guidelines

Session 3 - Practice Patient-Provider Interactions

Session 4 - Health Equity

Session 5 - Graduate Applications Walkthrough

Session 6 - Occupation Walkthroughs

Program Recommendation

	Not at all recommend				Slightly recommend				Highly recommend		
	0	1	2	3	4	5	6	7	8	9	10
On a scale of 1 to 10, would you recommend this program to peers in pre-health studies at ECU?											

Please share with us any additional comments, suggestions, or ideas for future sessions.

Powered by Qualtrics
